# Seed glucosinolate yield is maximized by higher rates of sulfur nutrition than required for seed yield in condiment mustard (*Brassica juncea* L.)

**DOI:** 10.1371/journal.pone.0213429

**Published:** 2019-04-02

**Authors:** Priyakshee Borpatragohain, Terry J. Rose, Lei Liu, Carolyn A. Raymond, Bronwyn J. Barkla, Graham J. King

**Affiliations:** Southern Cross Plant Science, Southern Cross University, Lismore, New South Wales, Australia; Chungnam National University, REPUBLIC OF KOREA

## Abstract

Brassica crops require high amounts of inorganic sulfur (S) for optimum yield, and are characterized by the synthesis of S-rich glucosinolates (GSL). Although it is well established that seed and GSL yield can be increased by S fertilizer, the detailed relationship between S supply as primary source and the harvestable sinks of seed GSL and storage proteins is poorly understood. We tested the hypothesis that *Brassica juncea* mustard seed acts as a secondary S sink, and so require a higher rate of S to achieve maximum seed GSL compared to rates required to attain maximum seed biomass. Our experimental strategy involved comparing responses to available S for seed biomass, GSL, and protein. This was carried out in a protected environment using sand culture for a high-GSL condiment-type homozygous *B*. *juncea* genotype. A low-GSL canola-type was used as a control, in order to establish a base-line of response. Significantly more S was required to achieve maximum seed GSL than was required to achieve maximum seed mass. Total seed protein content was not significantly affected by increased S. The high-GSL line appeared to have an efficient mechanism of S supply to the secondary S sink, given the observed increase in seed S with increased S availability. From a practical point of view, increases in seed GSL with S availability suggests that S fertilizer rates should be optimized for maximum seed GSL yield, rather that optimizing for seed yield, as occurs for most other crops.

## Introduction

Oleiferous brassicas such as canola (*Brassica napus*), Indian mustard and condiment mustard (*B*. *juncea*) and Chinese cabbage, sarson and Indian rapeseed (*B*. *rapa)* require high amounts of inorganic sulfur (S) supply for optimum yield. This can be up to 5–8 times the amount required for wheat [1, 2]. Most inorganic S in the mature seed of brassica is sequestered in the storage proteins cruciferin and napin, and in the secondary metabolite glucosinolate (GSL) [3, 4]. GSLs have a wide range of beneficial effects in crop production and plant defense, with some contributing positively to human nutrition, such as the anti-tumorigenic 4 carbon (C_4_) side-chain aliphatic GSL glucoraphanin found in broccoli (*B*. *oleracea* var. italica) [5, 6]. In contrast, anti-nutritional effects of GSLs on livestock [7] have led to the secondary domestication and widespread cultivation of canola-type rapeseed containing low seed C_3_ and C_4_ side-chain aliphatic GSLs. Canola now plays an important role in cereal rotations of global temperate arable systems.

Brassica seed contains high quality vegetable protein with balanced amino acid composition [8]. Compared with other grains such as soybean, the composition of brassica seed protein is closer to FAO recommendations for humans, having 3–4% of S-containing amino acids [9, 10]. Brassica seed storage proteins typically represent up to 30% of seed mass [11, 12] with 60% of the total protein represented by globulin-like cruciferins (cru) and 20% by 2S albumin-type napins (nap) [13]. Interestingly, a close negative correlation has been detected in *B*. *napus* between seed storage protein (cru/nap) ratio and GSL content [3]. However, the allergenicity of napins in humans [14] continues to limit the use of protein-rich brassica seed meal.

The interaction between S availability and the S-rich GSL secondary metabolites has long drawn the attention of agronomists and plant breeders. We recently reviewed the current understanding of the many underlying mechanisms that affect the interaction between S availability and seed GSL concentration [15]. Much of this knowledge has improved in recent years based on studies in the model plant *Arabidopsis thaliana* (Brassicaceae), as well as in *Brassica* crop species [16–18]. Inorganic sulfate, taken up by the plant, is reduced to organic S forms that include amino acids, glutathione, chloroplast lipids and GSLs, through S assimilation processes [19], with up to 8% of total plant S stored within GSL molecules [16]. Based on molecular interactions between sulfate and GSL transporters, transcription factors and signaling molecules, we developed a provisional model to describe the key processes that could be targeted in crop breeding by focusing on modifying GSL and protein content [15]. Our analysis indicated that the inherent genome complexity of *Brassica* species could play a major role in the regulation of S and GSLs, due to gene duplication and subsequent divergence driving ontogenetic plasticity during crop development.

In order to build on this conceptual framework a detailed understanding of the distribution and remobilization of S and GSLs throughout *Brassica* crop development is required. By representing this in terms of primary and secondary sources and sinks for S and GSLs, there is scope to account for S flux in the secondary seed sink between the S components of storage proteins and GSL [15]. Capitalising on the tendency of *Brassica* genotypes selected for low seed GSL content to have mutations in genes associated with specific GSL synthesis enzymes [20, 21], it should be possible to generate diagnostic evidence about specific seed S sinks, and distinguish this from transporter or remobilization signals from primary or secondary S sources [15].

In contrast to canola (*Brassica napus*, an allopolyploid comprised of A and C diploid genomes), higher levels of GSLs are often positively selected for their desirable culinary properties in the seeds of Indian mustard (*B*. *juncea* AB genome). This is possible due to the accumulation of the B-genome (*B*. *nigra*) derived C_3_ side-chain aliphatic GSL-sinigrin [22]. Indian mustard is widely grown within its center of genetic diversity India and elsewhere as an oilseed crop, and also as a condiment mustard in other parts of the world. In Australia, three different types of *B*. *juncea* (*juncea*-canola, condiment mustard and industrial mustard) are grown as high-value crops for production of canola-quality oil and meal, condiment mustard and volatile-mustard-oil (VOM), respectively [23].

In this study we test the hypothesis that within a, *B*. *juncea* line selected for high seed GSL concentration, the seeds act as a secondary S sink, and so plants require higher rates of S fertilizer (primary S source) to achieve maximum seed GSL yield compared to the rates required to attain maximum seed biomass and protein yield. We also test the hypothesis that the higher seed GSL yield associated with additional S supply results specifically from increases in sinigrin accumulation. Our experimental strategy involved comparing the response to applied S for seed biomass, GSL, and total protein yield. This was carried out in a protected environment using sand culture for a high-GSL condiment type homozygous *B*. *juncea* genotypes. We used a low-GSL canola-type genotype as a control, in order to establish a base line of response.

## Materials and methods

### Experimental design

A greenhouse trial was established at Southern Cross University (SCU), Lismore, Australia (28.8° S, 153.3° E) to determine plant biomass, seed biomass and GSL, along with seed protein yield responses to S fertilizer in high- and low-GSL mustard lines. Two *B*. *juncea* homozygous inbred lines differing in seed GSL concentrations, a high-GSL containing condiment-type *B*. *juncea* line O1493 and a low-GSL containing canola-type *B*. *juncea* line, C671 (sourced from Agriculture and Agri-Food Canada), were evaluated under 10 S fertilizer rate treatments to derive S response functions. The breeding pedigree of C671 indicates that the low-GSL trait was inherited via progenitor lines traceable to the original *B*. *napus* cv. Bronowski source [24, 25] and carried on the A genome with extensive introgression into *B*. *juncea*. Overall morphology was similar between the lines, including similar bivalve silique structures and size. The experiment was set out in a randomized block design with two mustard lines and 10 S fertilizer treatments replicated five times.

### Growth conditions

We conducted a series of preliminary experiments to optimize the growing medium and limit the set of tested S fertilizer rates to the responsive range. The lowest S fertilizer rate selected from the preliminary experiment was the minimum rate that enabled plants to reach maturity and form viable seed.

Plants were grown in 15-cm-diameter, free-draining plastic pots filled with approximately 2 kg of dried, washed river sand. On 6^th^ May 2016, three seeds were sown 5mm deep in each pot and thinned to one healthy seedling 12 days after emergence. Pots were flushed through daily with 0.5 L of nutrient solution until harvest. The basal nutrient solutions used in the experiment contained (μM): 4000 N (as NH_4_NO_3_), 500 K and 500 P (as KH_2_PO_4_), 1000 Ca (as CaCl_2_.2H_2_O), 500 Mg (as MgCl_2_), 9.8 B (as H_3_BO_3_), 2 Mn (MnSO_4_·H_2_O), 2 Zn (ZnSO_4_·7H_2_O), 0.5 Cu (CuSO_4_·5H_2_O), and 0.08 Mo (Na_2_MoO_4_·2H_2_O). For the 10 different S treatments, the basal nutrient solution was modified with (μM): 75, 100, 125, 150, 200, 300, 400, 500, 750 and 1000 S (as K_2_SO_4_) and KCl (1850, 1800, 1750, 1700, 1600, 1400, 1200, 1000, 500 and 0, respectively, to equalize K additions across the 10 S rates).

Temperatures inside the greenhouse during the experiment ranged from 8.4 to 29.5°C. Supplementary lighting (600 w HPS lamps for 12–16 hrs.) was used to initiate the flowering process at 45 days after sowing and removed after 50% flowering. Plants were harvested when all plants had reached physiological maturity, which occurred at 133 days after sowing for each line.

### Measurements and chemical analysis

After harvest, seed and stalk straw were dried in an air-forced oven at 40°C for 72 hrs, weighed and ground separately using a laboratory ball mill (Mixer Mill MM301, Retsch) for subsequent analyses. Seeds were ground with an equal amount of cellulose to achieve a homogenous mixture to mitigate the presence of oil in the seed.

Sample preparation and extraction of GSL from the seeds followed a modified procedure of Tian *et al*., (2005) and Crocoll *et al*., (2016) [26, 27]. In brief, each ground seed sample (~15 mg, with cellulose) or single seed (~3 mg, without cellulose) (used only for plants with very low yield) was extracted with 1.5 mL of 70% aqueous methanol in 2 mL Eppendorf safe lock microcentrifuge tubes. To achieve a homogenous mixture, tubes were shaken at 30 rotation s^-1^ for 30 s using a Qiagen Retsch TissueLyser II. The extracts were centrifuged using a Sigma lab table top centrifuge at 15,000 rpm for 15 min at 7°C. Subsequently, a 0.5 mL aliquot of each extract was transferred to a 2 mL Agilent HPLC screw cap vial and dried under nitrogen gas. The dried samples were reconstituted in 1 mL deionised water containing 1.17μmol mL^-1^ glucotropaeolin (internal standard) and sonicated for 10 min before Liquid Chromatography-Mass Spectrometry (LC-MS) analysis.

All extracts were analyzed using an Agilent 1290 High Performance LC-MS instrument (Agilent Technologies, Palo Alto, CA, USA) equipped with an autoinjector, vacuum degasser, binary pump and diode array detector (DAD, 1260), coupled with an Agilent 6120 quadrupole mass selective detector (MSD). A Kinetex 2.6 μm EVO C18 reverse phase column (100 x 2.1 mm internal diameter) (Phenomenex, Torrance, CA, USA) was used, with temperature set at 30°C. A linear gradient elution program was applied consisting of a mobile phase containing Milli-Q water with 0.01% trifluoroacetic acid (TFA) (solvent A) and acetonitrile with 0.005% TFA (solvent B) at a flow rate of 0.3 mL/min and 5μL injection volume. The 18 min run consisted of 0% B (8 min), 25% B (10 min), 100% B (13 min) and 0% B (18 min). The MSD was operated in atmospheric pressure ionization-electrospray (API-ES) mode with the following parameters: fragmentor, 150; capillary voltage, 3000 V (negative); drying gas flow, 12 L/min (N_2_); vaporizer temperature, 350 °C; nebulizer pressure, 35 psi; drying gas temperature, 350 °C. Absorbance was monitored at 210, 280 and 360 nm. Single ion monitoring (SIM) mode was set to detect 7 ions simultaneously in negative ion mode using four available mass selective detection signal channels such as signal 1: sinigrin (SIN) at *m/z* ratio of 358 for 0–8 min and glucotropaeolin (GTP) at *m/z* ratio of 408 for 8 to 18 min, signal2: progoitrin (PGT) and epiprogoitrin (EPI) at *m/z* ratio of 388 for 0–18 min, signal 3: glucoiberin (GIB) at *m/z* ratio of 422 for 0–10 min and gluconasturtiin (GNT) at m/z ratio of 422 for 10–18 min, signal 4: gluconapin (GNP) at *m/z* ratio of 372 for 0–18 min. Glucotropaeolin (GTP), not found in brassicas, was used as the internal standard to monitor the performance of MS [28]. All LC-MS settings and parameters above were optimized based on the manufacturer’s recommendations and a number of flow injection experiments. All the organic solvents used in the analysis were HPLC or LC-MS grade. Commercial GSL standards were obtained from PhytoLab GmbH & Co. KG, Germany.

Protein concentration in the seed was determined by the Bradford protein assay [29] using Bovine Serum Albumin (BSA) as a standard. Ground seed samples (~10 mg) were dissolved in Milli-Q water (600 μL) and shaken at 15 rotation s^-1^ for 15 s using a Qiagen Retsch MM 301 TissueLyser II, followed by 1 hr sonication in dry ice prior to protein estimation to achieve a homogeneous mixture. The Bradford assay reaction mixture consisted of 2 μL homogenized seed sample, 30 μL 0.05% Triton, 800 μL Milli-Q water and 200 μL Bradford dye reagent (BioRad). Samples were prepared in 1.5 mL microcentrifuge tubes and were vortexed after addition of each component to the reaction mixture and the mixture was incubated for 5 min after addition of Bradford dye. Subsequently, 200 μL of reaction mixture of each sample was loaded into a flat-bottom polystyrene clear 96 well microplate (Greiner Bio-One International) along with blank and five sets of BSA standards. Each sample, blank and standard was replicated twice and absorbance at 595 nm determined using a BMG Labtech ClarioStar microplate reader. The concentration of protein (mg g^-1^ of seed) in the sample was calculated by blank corrected linear regression fit of the BSA standard curve.

Concentrations of S (mg g^-1^) in the seed and stalk straw were determined using a LECO CS combustion analyzer at the Environmental Analysis Laboratory, SCU, Lismore, Australia. Sulfur accumulation in the seed and stalk straw (mg plant^-1^) was calculated by multiplying the S concentration (mg g^-1^) with respective biomass yield (g plant^-1^).

Seed GSL and protein yield (mg plant^-1^) were calculated by multiplying the seed GSL and seed protein concentration (mg g^-1^ of seed) with the seed biomass yield (g plant^-1^).

### Statistical analyses

Data analysis was undertaken using Genstat 64-bit Release 18.1 (VSN International Ltd.) software. After analyses of variance (ANOVA), the means of each observed trait from each S treatment for both high- and low-GSL line were compared using least significant difference (LSD) at P <0.05.

Data on average seed biomass yield, seed GSL and protein yield and total biomass yield response to applied S for both genotypes were fitted with a modified Mitscherlich function [30] described by the equation:

$$y=a-b\;e^{(-cx)n}$$ (1)

where *y* is the seed or total biomass or seed GSL or protein yield (g plant^-1^), *x* is the level of applied S (μM) and a,b,c and n are coefficients. The ‘a’ coefficient estimates the yield maximum (asymptote) as x approaches infinity, the ‘b’ coefficient estimates the difference between the asymptote and the intercept on the y-axis, and ‘c’ describes the shape of the response curve. The ‘n’ coefficient also affects the shape of the relationship: as the value of n increases above 1 (when n = 1 the response curve is exponential) the shape of the curve becomes increasingly sigmoidal [30]. Mitscherlich functions were fitted in Microsoft Excel using the ‘solver’ function.

The level of S (μM) required to achieve 90% seed total biomass, GSL and protein yield was calculated using the fitted equation and solved for *x* when *y* = 90% of level predicted for the maximum level of sulphur (1000 units). To test whether S rate for each trait was significantly different, we fitted the curves for each replicate and solved for S rate corresponding to 90% yield. The difference between traits for these *x* values was then tested by a one-way ANOVA with fitted trait values as the effect and using the variation amongst the individual plant values as the error term.

## Results

### Plant growth and phenology

The use of sand culture enabled the constant availability and control of nutrients throughout the cultivation of the plants. The set of 10 S concentrations covered the full response range for seed biomass, seed GSL and protein yield. At the lower S concentrations (75 to 150 μM) we observed yellowing of younger leaves in both lines, along with stunted growth indicating a deficiency. Flowering occurred from 60 to 65 days after sowing in both lines and was unaffected by S treatment.

### Total biomass, seed, glucosinolate and protein yield response to applied sulfur levels

**Fig 1:**
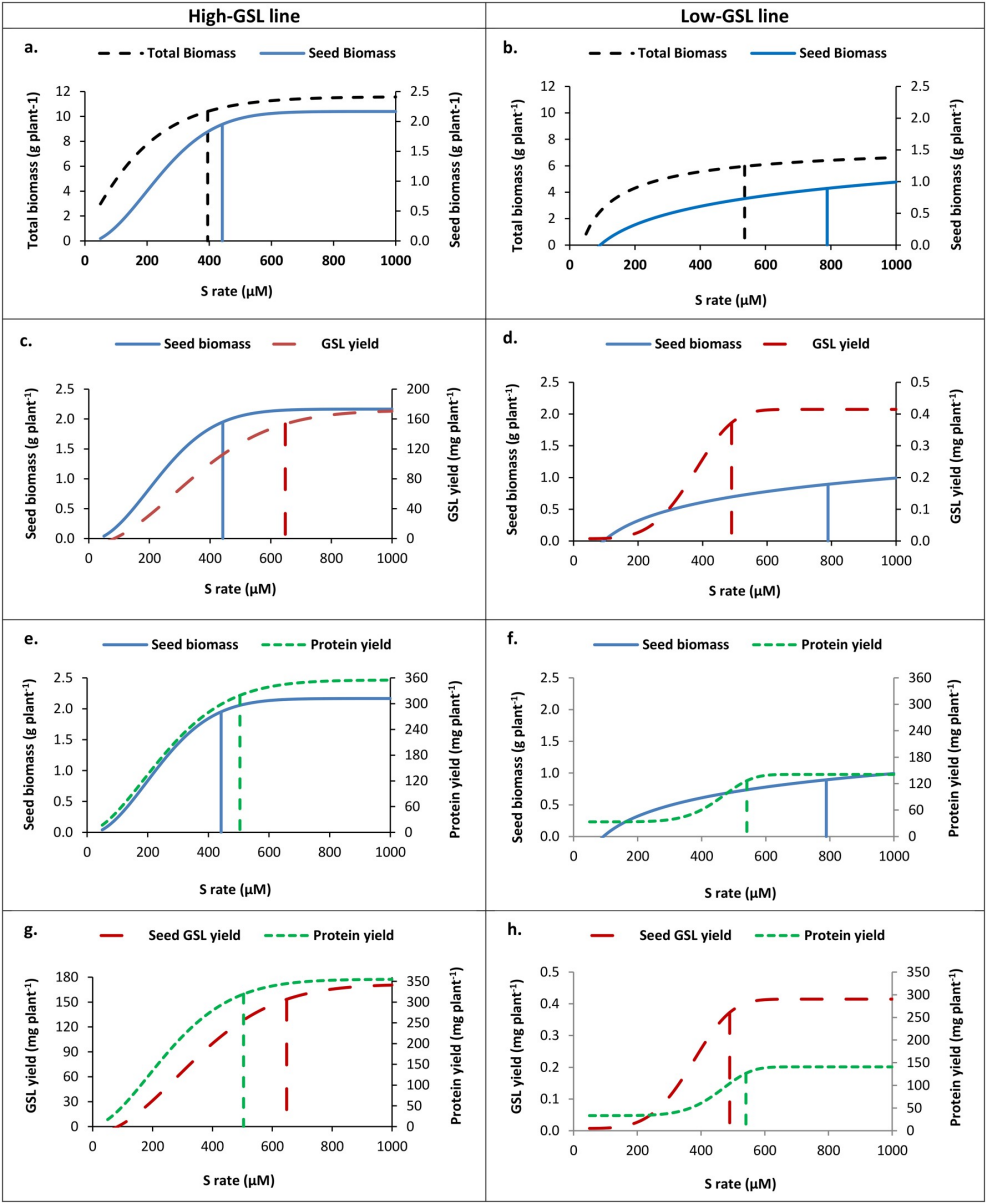
Fitted sulfur response curves using a modified Mitscherlich function with average trait data (S1 Table). Seed and total biomass yield in high- (a) and low-GSL (b) lines, seed yield and seed GSL yield in high- (c) and low-GSL (d) lines, seed yield and seed protein yield in high- (e) and low-GSL (f) lines and seed GSL and seed protein yield in high- (g) and low-GSL (h) lines. Vertical lines indicate applied S level required for 90% predicted maximum respective seed biomass, total biomass, seed GSL and protein yield. Total biomass and seed biomass yield (a-f) are expressed as g plant^-1^. Seed GSL and seed protein yield (c-h) are expressed as mg plant^-1^.

Both high- and low-GSL lines responded to S with an increase in total biomass, seed biomass, seed GSL and seed protein yield (S1 Fig). Maximum predicted total biomass (above ground parts) yield was around 10 g plant^-1^ in the high-GSL line, with a concentration of ~395 μM S in the nutrient solution required to achieve 90% maximum biomass yield (Fig 1a). In contrast, the maximum predicted total biomass yield for the low-GSL line was half that of the high-GSL line, and required 536 μM S (36% more) in the nutrient solution to achieve 90% maximum yield (Fig 1b).

Maximum predicted seed biomass yield was 2 g plant^-1^ for the high-GSL line, and 1 g for the low-GSL line (Fig 1a and 1b). The S concentration in the nutrient solution required to achieve 90% maximum seed yield was 12% higher than that needed for maximum total biomass yield in the high-GSL line, and 47% higher than that needed for maximum total biomass yield in the low GSL line (Table 1).

**Table 1:** Predicted values for 90% of maximum yield per plant for each trait, and predicted sulfur required to achieve this yield maxima, based on average trait data solved using modified Mitscherlich function.

Traits	High-GSL line		Low-GSL line	
	90% yield max. (mg per plant)	Sulfur level (μM) at 90% yield max	90% yield max. (mg per plant)	Sulfur level (μM) at 90% yield max
Total biomass	10,404	395	5,958	536
Seed biomass	1,950	442	890	789
GSL	153	648	0.4	490
Protein	319	504	127	541
LSD (p<0.05)		170.6		192.2

Differences between traits within each line were tested for significance p<0.05 by using LSD.

Seed GSL yield in the low-GSL line was low, with a predicted maximum seed GSL yield of 0.38 mg plant^-1^ (Fig 1d). The fitted model using Mitscherlich function indicated that a concentration of 490 μM S was required in the nutrient solution to achieve 90% maximum seed GSL yield, which was 61% lower than the S required to achieve maximum seed yield. In contrast, seed GSL yield in the high-GSL line reached 153 mg plant^-1^, with a concentration of 648 μM S required in the nutrient solution to achieve 90% of the maximum seed GSL yield (Fig 1c). This was almost 47% higher than the S rate required for maximum seed yield (Table 1). The predicted S concentrations required to achieve 90% maximum seed GSL yield was significantly higher than that required for seed yield in high-GSL line (Table 1).

Maximum predicted seed protein yield in the high-GSL line reached up to 319 mg plant^-1^, whereas in the low-GSL line the maximum predicted protein yield was only 40% of this (126.6 mg) (Table 1). The S concentration required to achieve 90% maximum seed protein was significantly less (45%; P <0.05) than that required for seed yield in the low-GSL line (Fig 1f; Table 1). However, in the high-GSL line the S concentration in the nutrient solution required to achieve 90% maximum seed protein yield was not significantly different from maximum seed yield (Fig 1e).

Fitted S response curves of seed GSL yield against seed protein yield for the low-GSL line showed that a higher S concentration (about 10%) could increase seed protein yields and had no effect on seed GSL (Fig 1h). In contrast, for the high-GSL line the fitted curves showed that higher S concentration (28%) led to higher seed GSL yield, rather than an increase in seed protein (Fig 1g).

### Response of seed glucosinolate concentrations and glucosinolate fractions to sulfur

Total seed GSL concentration for the high-GSL line increased from 25 to 208 μmol g^-1^ with increased supply of S (S2 Table). As expected, C_3_ side-chain aliphatic GSL-sinigrin was the major GSL fraction in this line, accounting for 99.3% of the total detected GSLs regardless of S treatment (S3 Table). The relative proportions of the minor C_4_ side-chain aliphatic GSL-progoitrin and gluconapin, and also the aromatic GSL-gluconasturtiin, were signifincatly affected by S supply (S2 Table). However, these values do not make a significant contribution to the seed S sink, and are of limited practical relevance given that they represent <1% of total seed GSL content.

The low-GSL line had only 0.3% of the seed GSL content of the high-GSL line. Although the C_4_-gluconapin proportion of total GSLs found in the low-GSL line was significantly affected by S treatment, such changes are of limited relevance since the total concentration of GSLs in the seeds of this line was only 0.03–1.7 μmol g^-1^ of seed (S3 Table).

### Effect of sulfur level on aboveground sulfur accumulation and partitioning

**Fig 2:**
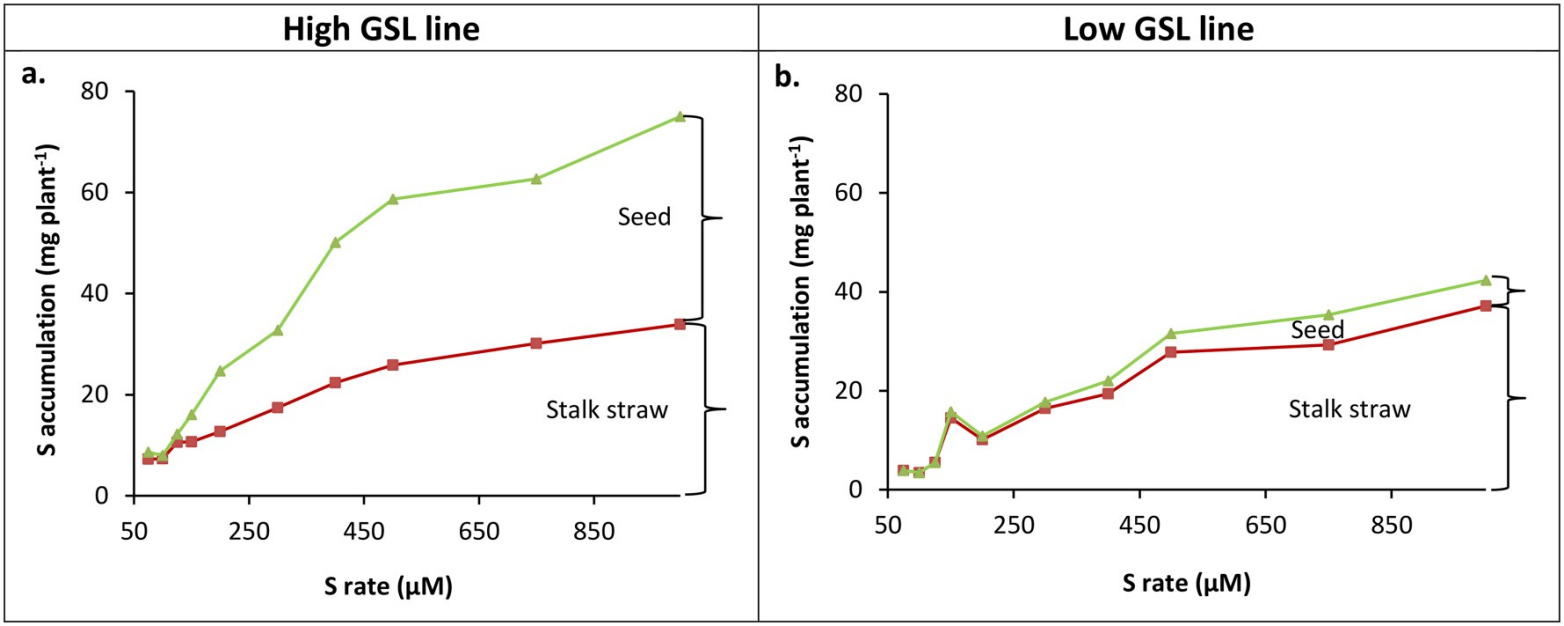
Effect of sulfur supply on partitioning of sulfur between seed and stalk straw. (a) the high-GSL line and (b) the low-GSL line. Combined S (mg plant^-1^) accumulation of seed and stalk straw are shown by green lines and only stalk straw are shown by red lines.

The accumulation of S in the seed and stalk straw of both low- and high-GSL lines generally increased with increased S supply (Fig 2). In the low-GSL line, seed S concentration significantly increased only up to 400 μM S in the nutrient solution, with no further increase at higher S levels (Table 2). For the high-GSL line, there was a consistent significant increase of seed S concentration with higher S supply up to the highest S level (1000 μM) (Table 2).

**Table 2:** Sulfur concentrations in the seed and stalk straw in response to sulfur levels (low = low-GSL line and high = high-GSL line).

S rate	Seed S concentration (mg g^-1^)		Stalk straw S concentration (mg g^-1^)	
	Low	High	Low	High
S _75_	0.00	5.92	1.79	1.74
S_100_	0.00	7.47	2.16	1.72
S_125_	0.00	8.46	1.68	1.98
S_150_	2.92	10.21	3.03	1.82
S_200_	3.69	10.94	2.87	1.71
S_300_	4.24	13.44	3.81	2.18
S_400_	5.07	14.76	4.37	2.69
S_500_	5.12	15.12	4.92	2.89
S_750_	5.54	16.21	4.95	3.20
S_1000_	5.51	18.53	7.23	3.62
LSD (p<0.05)	0.42	3.17	0.97	0.30

Differences between the means were tested for significance at p <0.05 by using LSD.

The S accumulated in the seeds of the high-GSL line was 5.3 times higher than that of the low-GSL line, irrespective of S level in the nutrient solution (S4 Table). However, S content of the stalk straw was similar in both lines (Fig 2.)

## Discussion

The majority of inorganic S (as sulfate) taken up by the roots (secondary S source) of Brassicaceae is transported to shoots (primary S sink), where it undergoes enzymatic reduction to organic S forms that include glutathione, cysteine, methionine and PAPS (3'-phosphoadenosine 5'-phosphosulphate) [19]. These assimilated S forms produced in the primary S sink at an early stage of plant development may later act as a S source for primary and secondary GSL sinks, such as siliques and seeds [31]. However, a complete picture of how S source and sink distribution changes over crop developmental stages has not been fully resolved [15]. We previously developed a model of the complex network of transporters, signaling molecules and transcription factors regulating S-metabolism in the context of source-sink relationship within brassicas. This suggested that the mature seed embryo acts as the ultimate sink for S-containing metabolites [15].

Despite the extensive literature addressing the effect of S on seed yield and GSL levels in canola (*B*. *napus*), few studies have investigated interactions between S, seed yield, GSL levels and protein content in mustard (*B*. *juncea*). Historically, a key driver for this research has been the need to optimize S fertilizer rates in canola crops to meet market specifications of maximized seed yields and minimal seed GSL [18]. In one of the few S fertilizer rate studies on canola [32], a mustard line was used as a comparative species. This study suggested that GSL yield (i.e. seed yield x seed GSL concentration) for the mustard, but not canola, continued to increase at S fertilizer application rates greater than those needed to obtain maximum seed yield. However, the levels of GSL and protein in the mustard line in response to S received no attention in the discussion. Thus, it was not known whether the observed increases in total GSL level were due to increases in the economically valuable C_3_ aliphatic-sinigrin, or other GSLs.

In the current study, we tested the hypothesis that the seed of mustard cultivars selected for high GSL concentrations act as a secondary S sink, and so require higher rates of S fertilizer as the primary S source to achieve maximum seed GSL yield than is required to achieve maximum seed biomass and protein yield. Our results indicated that while a seed biomass yield plateau was reached at a S concentration of 442 μM, seed GSL yield in the high-GSL line continued to increase significantly up to 648 μM S (Fig 1c and Table 1). This suggests that seed GSL acts as a secondary S pool in the high-GSL line. It is also consistent with the field trial data presented by Malhi *et al*., (2007) [32], where seed GSL concentrations (μmol g-1 of seed) appeared to continue to increase with S fertiliser rates above the 30 kg Sha-1 required to achieve maximum seed biomass yield. The higher S accumulation we observed in the seed of the high-GSL line indicates that this secondary S sink can efficiently use additional S supply (Fig 2a and Table 2).

We also tested the hypothesis that the higher seed GSL yield associated with additional S supply results specifically from an increase in C_3_ sinigrin accumulation. We found that C_3_ signigrin was the major (99.3%) GSL component in the high-GSL line, with C_4_ gluconapin and C_4_ progoitrin present only in trace amounts, consistent with previous reports in *B*. *juncea* [22, 33]. In contrast, C_4_ gluconapin was the predominant forms of GSLs in the seeds of low-GSL line with only trace levels of C_4_ sinigrinin. This may be due the presence of *B*. *napus* alleles in the low-GSL line. The diversity of GSL composition in *Brassica* species is associated with each of the A, B or C genomes. Three carbon side chain sinigrin found in *B*. *juncea* (AB genome) is attributed to the B genome (*B*. *nigra*) whereas four and five carbon GSLs are attributable to the A genome (*B*. *rapa*). This contrasts with three or four carbon side chain aliphatic GSLs present in *B*. *napus* (AC genome) attributable to the C genome (*B*. *oleracea*) [34]. Irrespective of S rates, the C_3_-signigrin fraction remained the major component in the high-GSL line, indicating that sinigrin is the key driver of S flux in the mature seed of condiment-type mustard cultivar (S2 and S3 Tables). The hydrolysed products of sinigrin, especially-ally-isothiocyanate (AITC), is also present in horseradish (*Armoracia rusticana)* and wasabi (*Eutrema japonicum*). AITC is commercially traded as volatile mustard oil, and can be used as a food flavoring agent, as a natural preservative to prevent the growth of certain fungi [35] and bacteria [36], and also as a biofumigant for soil borne pests [37]. Thus, increasing C_3_-sinigrin yield as opposed to other C_4_ and C_5_ aliphatic-GSLs is desirable for industrial condiment mustard.

As expected, the GSL yield of the low-GSL control line was marginal, only 0.3% of the high-GSL line, and reached a maximum at S levels below those required for maximum seed yield (Fig 1d and Table 1). This is consistent with reports that seed GSL concentrations do not continue to increase when S fertiliser rates above those required for maximum seed biomass yield are applied to canola-type *B*. *juncea* or *B*. *napus* cultivars [32, 38]. An increase in either the primary (soil) or secondary S source (stalk straw) was not able to increase the total S content in the seeds of the low-GSL line (Fig 2b and Table 2). Based on our understanding of S metabolism in Brassicaceae [15] this either could be due to reduction in activity or specificity of methylthioalkylmalate (MAM) synthase genes, or loss of GSL transporter function. In *B*. *napus*, mutations in MAM affect the side-chain elongation step of aliphatic GSL synthesis resulted in a low C_4_ and C_5_ side-chain aliphatic GSL phenotype [21]. In *A*. *thaliana*, mutation of GSL transporters eliminated GSL production, whilst mutation of a subset of orthologues in *B*. *rapa* and *B*. *juncea* reduced GSL content in the seed upto 60% [39].

## Conclusion

*Brassica juncea* homozygous condiment-type line with high-GSL content and low GSL canola-type line responded differently to increased S availability. The former required significantly higher S to achieve maximum seed GSL than that was S required for maximum seed mass. The high-GSL line appeared to have an efficient mechanism to supply S to the secondary S sink, given the observed increased in seed S with increased S availability. This contrasts with the apparent defect in either early GSL synthesis or in GSL transport in the low-GSL line. From a practical point of view, the increase in seed GSL with higher rates of S availability suggests that S fertilizer application rates in a given environment should be optimized for maximum seed GSL yield, rather that optimizing S rates for seed yield, as occurs for most other crops. These preliminary findings will be explored further in a population segregating for seed GSL content.

## Supporting information

S1 Fig Sulfur response curves for biomass, seed, glucosinolate and protein yield of high-and low-GSL line with original and fitted values following Mitcherlich function. Total biomass and seed biomass yields are expressed in g plant^-1^; seed glucosinolate and seed protein yield are expressed in mg plant^-1^.
(TIF)

S1 Table Average total biomass, seed biomass, seed GSL and seed protein yields of both low- and high-GSL lines at ten different rates of S supply. SD values were calculated from five biological replicates.
(PDF)

S2 Table Response of sulfur on total detected glucosinolate and each glucosinolate fractions (% of glucosinolate) of each sulfur treated tested for significance at p<0.05. (PDF)

S3 Table Mean GSL fractions (% of total detected GSLs by LC-MS) ± standard deviation for both low- and high-GSL line. The significance of differences (*p≤0.05, **p≤0.01) between sulfur levels in one way-ANOVA.
(PDF)

S4 Table Sulfur uptake by the seed and stalk straw in response to different levels of sulfur in low- and high-GSL line. Differences between means were tested for significance at p<0.05 by using LSD.
(PDF)

## References

[1] Anjum NA, Gill SS. Umar S, Duarte AC, and Pereira E, Improving Growth and Productivity of Oleiferous Brassicas under Changing Environment: Significance of Nitrogen and Sulphur Nutrition, and Underlying Mechanisms. The Scientific World Journal, 2012 2012: p. 657808 10.1100/2012/657808 22629181 PMC3353521

[2] Zhao FJ, Withers PJA, Evans EJ, Monaghan J, Salmon SE, Shewry PR et al, Sulphur nutrition: An important factor for the quality of wheat and rapeseed. Soil Science and Plant Nutrition, 1997 43(sup1): p. 1137–1142.

[3] Schatzki J, Ecke W, Becker HC, and Möllers C, Mapping of QTL for the seed storage proteins cruciferin and napin in a winter oilseed rape doubled haploid population and their inheritance in relation to other seed traits. Theoretical and Applied Genetics, 2014 127(5): p. 1213–1222. 10.1007/s00122-014-2292-0 24595811

[4] Lakkineni K, and Abrol Y, Effect of sulfur fertilization on Rapeseed-mustard and Groundnut. Phyton (Horn, Austria), 1992 32: p. 75–78.

[5] Bones AM, and Rossiter JT, The myrosinase-glucosinolate system, its organisation and biochemistry. Physiologia Plantarum, 1996 97(1): p. 194–208.

[6] Wittstock U, and Burow M, Glucosinolate Breakdown in Arabidopsis: Mechanism, Regulation and Biological Significanc. 2010: American Society of Plant Biologists. 10.1199/tab.0134 PMC3244901 22303260

[7] Griffiths D, Birch A, and Hillman J, Antinutritional compounds in the Brassicaceae: analysis, biosynthesis, chemistry and dietary effects. J. Hort. Sci, 1998 73(1): p. 1–18.

[8] Sosulski F, Organoleptic and nutritional effects of phenolic compounds on oilseed protein products: a review. Journal of the American Oil Chemists’ Society, 1979 56(8): p. 711–715.

[9] Ohlson R, and Anjou K, Rapeseed protein products. Journal of the American Oil Chemists’ Society, 1979 56(3): p. 431–437. 536560 10.1007/BF02671531

[10] Nishinari K, Fang Y, Guo S, and Phillips GO, Soy proteins: A review on composition, aggregation and emulsification. Food Hydrocolloids, 2014 39: p. 301–318.

[11] Dimov Z, Suprianto E, Hermann F, and Möllers C, Genetic variation for seed hull and fibre content in a collection of European winter oilseed rape material (*Brassica napus* L.) and development of NIRS calibrations. Plant breeding, 2012 131(3): p. 361–368.

[12] Nesi N, Delourme R, Brégeon M, Falentin C, and Renard M, Genetic and molecular approaches to improve nutritional value of *Brassica napus* L. seed. Comptes Rendus Biologies, 2008 331(10): p. 763–771. 10.1016/j.crvi.2008.07.018 18926490

[13] Crouch ML and Sussex IM, Development and storage-protein synthesis in *Brassica napus* L. embryos in vivo and in vitro. Planta, 1981 153(1): p. 64–74. 10.1007/BF00385319 24276708

[14] Monsalve R, Villalba M, and Rodríguez R, Allergy to mustard seeds: the importance of 2S albumins as food allergens. in Internet Symposium on Food Allergens. 2001.

[15] Borpatragohain P, Rose T, and King G, Fire and Brimstone: Molecular Interactions between Sulfur and Glucosinolate Biosynthesis in Model and Crop Brassicaceae. Front. Plant Sci., 2016 7(1735). 10.3389/fpls.2016.01735 PMC5116641 27917185

[16] Falk KL, Tokuhisa JG, and Gershenzon J, The Effect of Sulfur Nutrition on Plant Glucosinolate Content: Physiology and Molecular Mechanisms. Plant biol (Stuttg), 2007 9(05): p. 573–581. 17853357 10.1055/s-2007-965431

[17] Koprivova A, and Kopriva S, The importance of having sulfur. J Genet Genomics, 2016.

[18] Jankowski K, Budzyñski W, and Szymanowski A, Effect of sulfur on the quality of winter rape seeds. Journal of Elementology, 2008 13(4): p. 521–534.

[19] Mugford SG, Lee B, Koprivova A, Matthewman C, and Kopriva S, Control of sulfur partitioning between primary and secondary metabolism. The Plant Journal, 2011 65(1): p. 96–105. 10.1111/j.1365-313X.2010.04410.x 21175893

[20] Li G, and Quiros CF, Genetic analysis, expression and molecular characterization of BoGSL-ELONG, a major gene involved in the aliphatic glucosinolate pathway of *Brassica* species. Genetics, 2002 162(4): p. 1937–1943. 12524361 10.1093/genetics/162.4.1937 PMC1462373

[21] Liu Z, Hammerlindl J, Keller W, McVetty PBE, Daayf F, Quiros CF, et al, MAM gene silencing leads to the induction of C3 and reduction of C4 and C5 side-chain aliphatic glucosinolates in *Brassica napus*. Molecular Breeding, 2011 27(4): p. 467–478.

[22] Othmane M, Genetic Variability in Glucosinolates in Seed of *Brassica juncea*: Interest in Mustard Condiment. Journal of Chemistry, 2015 2015: p. 6.

[23] Haskins B, McCaffery D, and Bambach R, Juncea canola in the low rainfall zones of Victoria and South Australia, N.D.o.P. Industries, Editor. 2009.

[24] Love HK, Rakow G, Raney JP, and Downey RK, Development of low glucosinolate mustard. Canadian Journal of Plant Science, 1990 70(2): p. 419–424.

[25] Cheng BF, Séguin-Swartz G, Somers D, and Rakow G, Low glucosinolate *Brassica juncea* breeding line revealed to be nullisomic. Vol. 44 2001 738–41. 10.1139/g01-057 11550912

[26] Tian QG, Rosselot RA, and Schwartz SJ, Quantitative determination of intact glucosinolates in broccoli, broccoli sprouts, brussels sprouts, and cauliflower by high-performance liquid chromatography-electrospray ionization-tandem mass spectrometry. Analytical Biochemistry, 2005 343(1): p. 93–99. 10.1016/j.ab.2005.04.045 15963940

[27] Crocoll C, Halkier BA, and Burow M, Analysis and Quantification of Glucosinolates. Current Protocols in Plant Biology, 2016: p. 385–409. 10.1002/cppb.20027 30775863

[28] Fahey JW, Zalcmann AT, and Talalay P, The chemical diversity and distribution of glucosinolates and isothiocyanates among plants. Phytochemistry, 2001 56(1): p. 5–51. 11198818 10.1016/s0031-9422(00)00316-2

[29] Bradford MM, A rapid and sensitive method for the quantitation of microgram quantities of protein utilizing the principle of protein-dye binding. Anal Biochem, 1976 72: p. 248–54. 942051 10.1016/0003-2697(76)90527-3

[30] Barrow N and Mendoza R, Equations for describing sigmoid yield responses and their application to some phosphate responses by lupins and by subterranean clover. Nutrient Cycling in Agroecosystems, 1990 22(3): p. 181–188.

[31] Andersen TG and Halkier BA, Upon bolting the GTR1 and GTR2 transporters mediate transport of glucosinolates to the inflorescence rather than roots. Plant signaling & behavior, 2014 9(1): p. e27740. 24481282 10.4161/psb.27740 PMC4091225

[32] Malhi S, Gan Y, and Raney J, Yield, Seed Quality, and Sulfur Uptake of Oilseed Crops in Response to Sulfur Fertilization. Agronomy Journal, 2007 99(2): p. 570–577.

[33] Sodhi YS, Mukhopadhyay A, Arumugam N, Verma JK, Gupta V, Pental D, et al, Genetic analysis of total glucosinolate in crosses involving a high glucosinolate Indian variety and a low glucosinolate line of *Brassica juncea*. Plant Breeding, 2002 121(6): p. 508–511.

[34] Ishida M, Hara M, Fukino N, Kakizaki T, and Morimitsu Y, Glucosinolate metabolism, functionality and breeding for the improvement of Brassicaceae vegetables. Breeding Science, 2014 64(1): p. 48–59. 10.1270/jsbbs.64.48 24987290 PMC4031110

[35] Bednarek P, Piślewska-Bednarek M, Svatoš A, Schneider B, Doubský J, Mansurova M, et al, A glucosinolate metabolism pathway in living plant cells mediates broad-spectrum antifungal defense. Science, 2009 323(5910): p. 101–106. 10.1126/science.1163732 19095900

[36] Rhee MS, Lee S-Y, Dougherty RH, Kang D-H, Antimicrobial effects of mustard flour and acetic acid against Escherichia coli O157: H7, Listeria monocytogenes, and Salmonella enterica serovar Typhimurium. Applied and environmental microbiology, 2003 69(5): p. 2959–2963. 10.1128/AEM.69.5.2959-2963.2003 12732572 PMC154497

[37] Bellostas N, Sørensen J., and Sørensen H, Biofumigation: from the" classical" approach to the use of biorefined glucosinolates as natural plant protection agents. GCIRC Bulletin, 2007.

[38] Hassan F, Manaf A, Qadir G, and Basra SMA., Effects of sulphur on seed yield, oil, protein and glucosinolates of canola cultivars. International Journal of Agriculture and Biology (Pakistan), 2007.

[39] Nour-Eldin HH, Madsen SR, Engelen S, Jørgensen ME, Olsen CE, Andersen JS, et al, Reduction of antinutritional glucosinolates in Brassica oilseeds by mutation of genes encoding transporters. Nature Biotechnology, 2017. 10.1038/nbt.3823 28288105

