# Supplementary figures and images for: Seed glucosinolate yield is maximized by higher rates of sulfur nutrition than required for seed yield in condiment mustard (Brassica juncea L.)
Source: PLoS One. 2019 Apr 2;14(4):e0213429. doi: 10.1371/journal.pone.0213429 (PMC6445519; doi:10.1371/journal.pone.0213429)

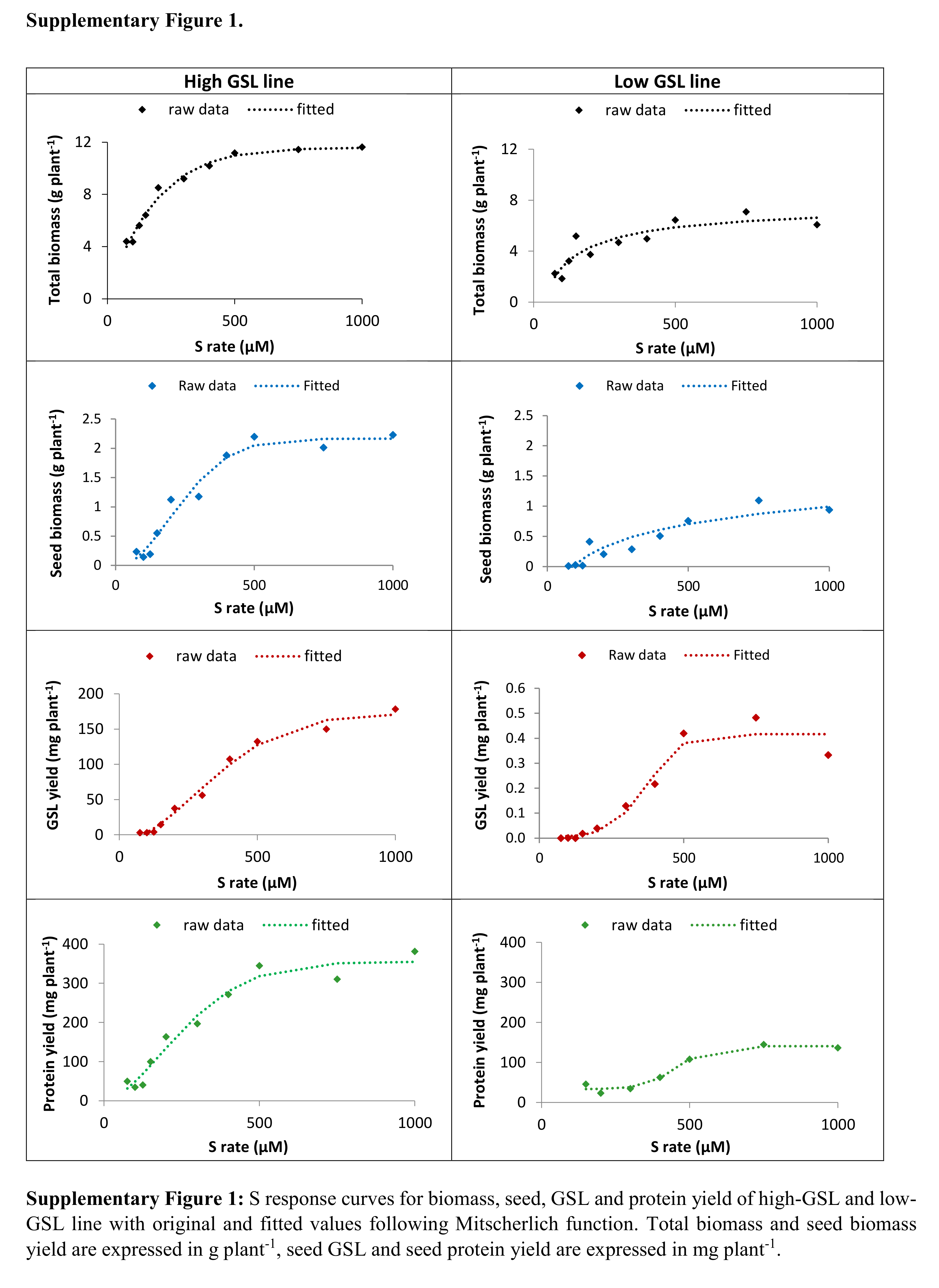

Supplement: S1 Fig — Total biomass and seed biomass yields are expressed in g plant-1; seed glucosinolate and seed protein yield are expressed in mg plant-1. (TIF) [file pone.0213429.s001.tif]
