# Supplementary material for: Seed glucosinolate yield is maximized by higher rates of sulfur nutrition than required for seed yield in condiment mustard (Brassica juncea L.)
Source: PLoS One. 2019 Apr 2;14(4):e0213429. doi: 10.1371/journal.pone.0213429 (PMC6445519; doi:10.1371/journal.pone.0213429)
Supplement: S1 Table — SD values were calculated from five biological replicates. (PDF) [file pone.0213429.s002.pdf]

Supplementary Table 1. Average total biomass and seed biomass, seed GSL and seed protein yields of both low- and high-GSL line at 10 different rates of S. SDs were calculated from five biological replicates.

S rate	Total biomass (g plant^{-1})		Seed biomass (g plant^{-1})		Seed GSL (mg plant^{-1})		Seed protein (mg plant^{-1})	
Line	Low	High	Low	High	Low	High	Low	High
S_{75}	2.2±1.0	4.4±0.3	0.0±0.0	0.2±0.2	0.0±0.0	2.9±4.2	0.0±0.0	49.8±11.3
S_{100}	1.8±1.2	4.4±1.2	0.0±0.0	0.1±0.1	0.0±0.0	2.9±1.4	0.0±0.0	34.9±4.2
S_{125}	3.2±0.8	5.6±0.9	0.0±0.0	0.2±0.1	0.0±0.0	3.9±3.0	0.0±0.0	40.7±3.8
S_{150}	5.2±1.0	6.4±0.6	0.4±0.2	0.6±0.2	0.0±0.0	14.1±4.2	45.8±30	100±36
S_{200}	3.7±1.4	8.5±0.9	0.2±0.1	1.1±0.3	0.0±0.0	37.3±9.1	23.5±14	164±50
S_{300}	4.7±1.0	9.2±1.2	0.3±0.1	1.2±0.4	0.1±0.1	56.1±11.3	34.6±15	197±85
S_{400}	5.0±2.3	10.2±0.4	0.5±0.3	1.9±0.1	0.2±0.2	107.1±8.1	63.1±36	218±41
S_{500}	6.4±0.9	11.2±0.8	0.8±0.2	2.2±0.5	0.4±0.4	132.1±20	108.4±33	345±42
S_{750}	7.1±0.4	11.4±0.5	1.1±0.1	2.0±0.4	0.5±0.2	150.0±26	145±13	311±60
S_{1000}	6.1±0.5	11.6±0.7	0.9±0.1	2.2±0.3	0.3±0.2	178.4±35	137±19	381±95
