# Supplementary material for: Seed glucosinolate yield is maximized by higher rates of sulfur nutrition than required for seed yield in condiment mustard (Brassica juncea L.)
Source: PLoS One. 2019 Apr 2;14(4):e0213429. doi: 10.1371/journal.pone.0213429 (PMC6445519; doi:10.1371/journal.pone.0213429)
Supplement: S2 Table — (PDF) [file pone.0213429.s003.pdf]

Supplementary Table 2.

Response of sulfur on total detected GSL and each GSL fractions (% of GSL) of each treatment tested for significance at p<0.05 by using LSD.

	Total detected GSL (μmol per g of seed)		% Sinigrin		% Progoitrin		% Epiprogoitrin		% Glucoiberine		% Gluconastrutiin		% Gluconapin	
Lines	Low	High	Low	High	Low	High	Low	High	Low	High	Low	High	Low	High
S_{75}	0.008	25.30	0.00	99.20	0.00	0.00	0.00	0.0000	0.00	0.03	0.00	0.010	0.00	0.68
S_{100}	0.039	41.20	0.00	99.22	0.00	0.00	0.00	0.0007	0.00	0.05	0.00	0.010	33.33	0.70
S_{125}	0.000	48.30	0.00	99.12	0.00	0.00	0.00	0.0000	0.00	0.14	0.00	0.004	0.00	0.73
S_{150}	0.071	73.81	0.00	99.21	3.87	0.00	0.00	0.0000	0.00	0.03	0.00	0.001	36.13	0.74
S_{200}	0.665	89.40	19.32	99.34	11.94	0.02	0.00	0.0000	0.00	0.03	0.10	0.009	68.64	0.59
S_{300}	1.208	131.52	1.62	99.37	6.02	0.01	0.00	0.0000	0.00	0.11	0.00	0.010	92.36	0.56
S_{400}	1.074	147.91	0.43	99.28	2.91	0.01	0.00	0.0000	0.00	0.16	0.00	0.050	96.65	0.54
S_{500}	1.751	159.22	3.38	99.26	3.44	0.01	0.00	0.0000	0.00	0.16	0.09	0.070	93.08	0.49
S_{750}	1.152	195.80	13.12	99.31	3.05	0.02	0.00	0.0000	0.00	0.12	0.00	0.050	83.82	0.48
S_{1000}	0.934	208.41	5.72	99.33	3.08	0.01	0.00	0.0000	0.00	0.11	0.00	0.050	91.19	0.47
LSD	0.703	19.18	14.33	0.15	4.10	0.0004	-	0.0002	-	0.08	0.09	0.010	23.33	0.09
