# Supplementary material for: Seed glucosinolate yield is maximized by higher rates of sulfur nutrition than required for seed yield in condiment mustard (Brassica juncea L.)
Source: PLoS One. 2019 Apr 2;14(4):e0213429. doi: 10.1371/journal.pone.0213429 (PMC6445519; doi:10.1371/journal.pone.0213429)
Supplement: S3 Table — The significance of differences (*p≤0.05, **p≤0.01) between sulfur levels in one way-ANOVA. (PDF) [file pone.0213429.s004.pdf]

Supplementary Table 3.

Mean GSL fractions (% of total detected GSLs by LC-MS) ± Standard Deviations for low and high-GSL lines. The significance of the differences (*p≤0.05, ** p≤0.01) between sulfur levels in one way ANOVA.

Glucosinolates	Low GSL line		High GSL line	
% Sinigrin	4.95 ± 14.33		99.27 ± 0.15	
% Progoitrin	3.901 ± 4.11		0.01 ± 0.004	**
% Epiprogoitrin	2.27 ± 12.31		0	
% Glucoiberine	0.00		0.08 ± 0.08	
% Gluconastrutiin	0.02 ± 0.09		0.03 ± 0.02	**
% Gluconapin	66.12 ± 23.33	**	0.60 ± 0.09	**
Total GSL concentration (μmolg^{-1})	1.7± 20.10	**	208± 0.06	**
