# Supplementary material for: Seed glucosinolate yield is maximized by higher rates of sulfur nutrition than required for seed yield in condiment mustard (Brassica juncea L.)
Source: PLoS One. 2019 Apr 2;14(4):e0213429. doi: 10.1371/journal.pone.0213429 (PMC6445519; doi:10.1371/journal.pone.0213429)
Supplement: S4 Table — Differences between means were tested for significance at p<0.05 by using LSD. (PDF) [file pone.0213429.s005.pdf]

Supplementary Table 4.

Sulfur uptake by the seed and stalk straw in response to different level of sulfur in low and high GSL cultivars. Differences between means were tested for significance at $p<0.05$ by using LSD.

S rate	S accumulated by seed (mg plant^{-1})		S accumulated by stalk straw (mg plant^{-1})	
Line	Low	High	Low	High
S_{75}	0.00	1.76	3.86	7.23
S_{100}	0.00	1.55	3.45	7.35
S_{125}	0.00	1.95	5.51	10.60
S_{150}	1.25	5.37	14.53	10.65
S_{200}	0.75	12.05	10.12	12.67
S_{300}	1.21	15.30	16.46	17.42
S_{400}	2.56	27.71	19.41	22.38
S_{500}	3.80	32.85	27.76	25.84
S_{750}	6.06	32.55	29.27	30.11
S_{1000}	5.19	41.14	37.14	33.89
Grand mean	2.98	18.95	16.75	17.82
LSD ($p<0.05$)	1.03	7.89	6.44	2.46
